# CD40 controls CXCR5-induced recruitment of myeloid-derived suppressor cells to gastric cancer

**DOI:** 10.18632/oncotarget.5644

**Published:** 2015-10-09

**Authors:** Yixin Ding, Jin Shen, Guangbo Zhang, Xiaojuan Chen, JiaMing Wu, Weichang Chen

**Affiliations:** ^1^ Departments of Gastroenterology, the First Affiliated Hospital of Soochow University, Suzhou, Jiangsu 215006, P.R. China; ^2^ Key Laboratory of Medicine and Clinical Immunology of Jiangsu Province, the First Affiliated Hospital of Soochow University, Suzhou, Jiangsu 215006, P.R. China

**Keywords:** myeloid-derived suppressor cells, CD40, CXCR5, gastric cancer, immune evasion

## Abstract

To explore the mechanisms of MDSC trafficking and accumulation during tumor progression. In this study, we report significant CD40 upregulation in tumor-infiltrating MDSC when compared with splenic MDSC. Microarray analyses comparing CD40^high^ and CD40^low^ MDSC revealed 1872 differentially expressed genes, including CD83, CXCR5, BTLA, CXCL9, TLR1, FLT3, NOD2 and CXCL10. *In vivo* experiments comparing wild-type (WT) and CD40 knockout (KO) mice demonstrated that CD40 critically regulates CXCR5 expression. Consistently, the transwell analysis confirmed the essential role of CXCR5-CXCL13 crosstalk in the migration of CD40^+^ MDSC toward gastric cancer. Furthermore, more MDSC accumulated in the gastric cancers of WT mice when compared with KO mice, and the WT tumors mostly contained CD40^+^ cells. Functionally, tumors grew faster in WT than KO mice. In conclusion, we demonstrate that CD40 expression upregulates the chemokine receptor CXCR5 and promotes MDSC migration toward and accumulation within cancer. Therefore, this study provides preliminary evidence that CD40 may stimulate tumor growth by enabling immune evasion via MDSC recruitment and inhibition of T cell expansion.

## INTRODUCTION

Gr-1^+^CD11b^+^ myeloid-derived suppressor cells (MDSC) are a heterogeneous population of immature cells that are capable of inducing a remarkable inhibition of the T-cell response [1]. The MDSC population includes macrophages, granulocytes, dendritic cells and other myeloid progenitor cells [1]. Clinical and experimental research has demonstrated that MDSC infiltrate tumors and promote tumor progression through the activation or production of L-arginine, inducible nitric oxide synthase (iNOS), reactive oxygen species (ROS), prostaglandin E2 (PGE2) and transforming growth factor β (TGFβ) [2–4].

Tumor-infiltrating MDSC primarily include bone-marrow-derived CD11b^+^Gr-1^hi^Ly-6C^int^ neutrophils and CD11b^+^Gr-1^int/dull^Ly-6C^hi^ macrophages [5]. The recruitment of these MDSC subtypes to tumor sites is mediated by chemokines highly expressed by tumor-associated neutrophils, including CXCR2 and CXCR4, and chemokines over-expressed by tumor infiltrating macrophages, including CCR2, CCR5, CXCR4 and CX3CR1 [5]. Studies have consistently demonstrated the critical role of CCR2 in the recruitment of monocytes to inflammatory sites [6–8], as well as the involvement of CCR5 and CX3CR1 in this process [9, 10]. In one study examining a type II TGFβ receptor (Tgfbr2) deletion mouse model of breast carcinoma, CD11b^+^Gr-1^+^ MDSC recruitment depended on the CXCL5/CXCR2 and SDF-1/CXCR4 chemotaxis axes [11]. It remains unclear whether the specific chemokines or chemokine receptor axes involved in MDSC recruitment are determined by tumor type, tumor stage or MDSC subset.

A number of recent studies have reported the importance of CD40^+^ MDSC in tumor growth. CD40 is a cell-surface co-stimulatory molecule that activates immune cells and immune responses through interaction with its ligand, CD154 [12–14]. CD40 promotes the recruitment of monocytes, lymphocytes and neutrophils to inflammatory tissues by stimulating the secretion of multiple chemokines from macrophages [15]. CD40 is expressed at high levels during tumorigenesis by tumor infiltrating MDSC [17]. MDSC expressed CD40 modulates MDSC-induced immunosuppression by interacting with T cell CD154 and inhibiting T-cell proliferation, upregulating PD-1 expression on CD4^+^ T cells, promoting the secretion of immunosuppressive cytokines such as TGFβ and IL-10 and stimulating the expansion of regulatory T cells (Treg) [16–18]. However, it remains unknown whether or not CD40 contributes to the trafficking and recruitment of MDSC. In this study, we examined the expression profile of CD40^+^ MDSC and investigated its potential involvement in MDSC recruitment to gastric cancer.

## RESULTS

### Tumor-infiltrating MDSC express high levels of CD40

**Figure 1:**
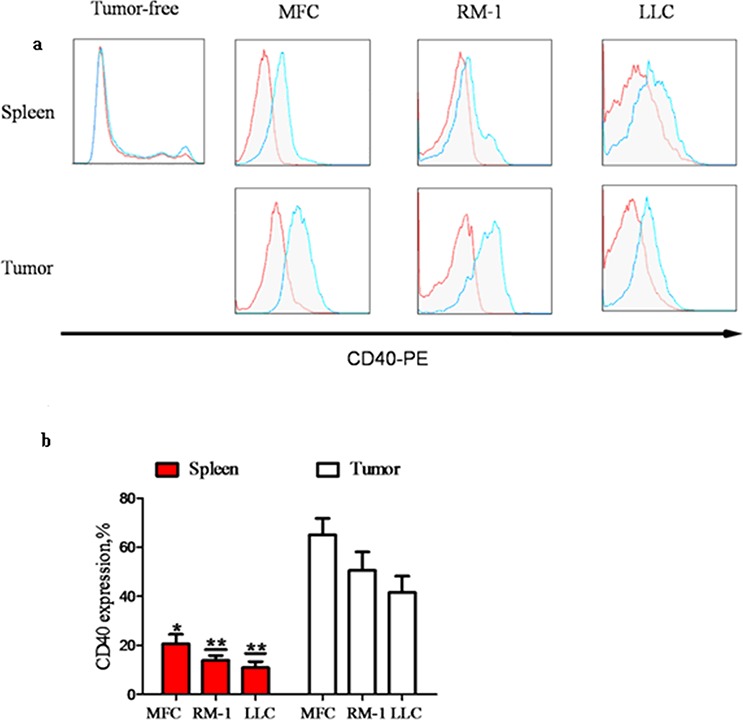
The percentage of CD40^+^ (CD40^+^%) MDSC was significantly elevated in mouse spleens after tumor formation and was significantly higher in tumor tissue when compared with splenic tissue MDSC were isolated from the spleens of WT C57BL/6 mice without tumor inoculation (tumor-free; *n* = 5) or from the spleens and tumors of mice with tumors (diameter = 1 cm; *n* = 5) grown from subcutaneously injected MFC, RM-1 or LLC cells. CD40^+^% MDSC were analyzed using flow cytometry after staining isolated MDSC with PE-conjugated anti-CD40 antibody (CD40^−^PE; blue line) or PE-conjugated isotype-matched IgG control antibody (red line). **a.** Representative flow cytometry images showing minimal CD40^+^ cell detection in tumor-free mouse spleen, and a dramatic increase in CD40^+^% MDSC after tumor formation from all three different types of cancer cells. The highest CD40^+^% levels were detected in tumor tissues. **b.** CD40^+^% quantification in different groups of mice. *p < 0.05 and **p < 0.01, compared to the corresponding tumor tissues.

To investigate the role of CD40 in MDSC cancer tissue infiltration, we established xenograft models using three different types of cancer cells: MFC for gastric cancer, LLC for lung cancer and RM-1 for prostate cancer. MDSC were isolated from the tumor tissues and spleens of xenograft mice and the spleens of control WT mice without tumor cell inoculation (tumor-free). Using flow cytometry analysis, we found that the percentage of CD40^+^ cells (CD40^+^%) in the splenic tissue of tumor-bearing mice (20.64 ± 3.84%, 13.86 ± 1.95% and 10.92 ± 2.45% from MFC-, LLC- and RM-1-injected mice, respectively) was significantly higher than the CD40^+^% in tumor-free WT mice (4.61 ± 0.34%; *p* < 0.05). Furthermore, the specific CD40^+^% for MDSC in tumor tissues (65.04% ± 6.71%, 50.56% ± 7.52% and 41.56% ± 6.69% from MFC-, LLC- and RM-1-injected mouse tumors, respectively) was significantly higher than the in the CD40^+^% in the spleen of the same mouse (*p* < 0.05; Figure 1). This suggested that the recruitment and accumulation of CD40^+^MDSC in tumor tissue is not unique to a specific cancer type. Because the most robust CD40^+^% difference was observed between MFC-formed tumors and the corresponding splenic tissues of the same animal, we focused on MFC-derived tumors for the subsequent experiments.

### CD40^high^ and CD40^low^ MDSC presented distinct gene expression profiles

**Figure 2:**
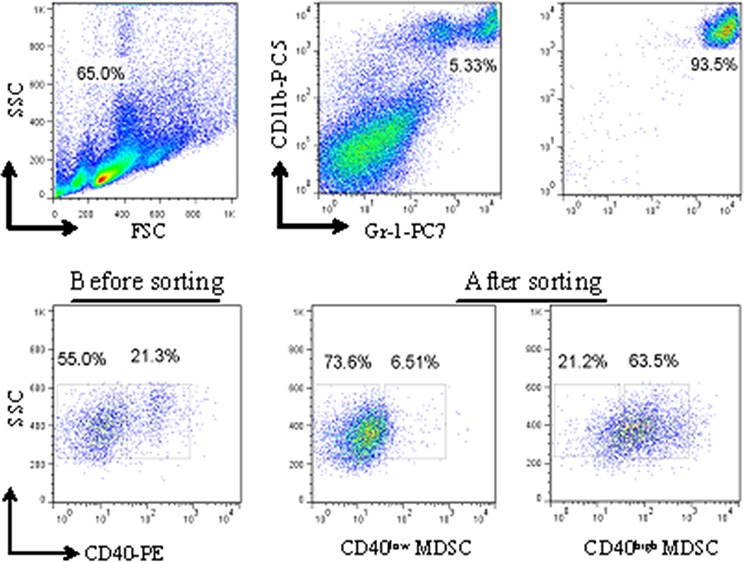
Isolation of CD40^high^ and CD40^low^ MDSC from MFC tumors by fluorescence-activated cell sorting (FACS) Single cells dissociated from MFC tumors were stained with fluorophore-conjugated anti-CD11b, anti-Gr-1 and anti-CD40 antibodies. The gating strategy for FACS as well as the pre- and post-sorting percentages of CD40^high^ and CD40^low^ MDSC are shown.

**Figure 3:**
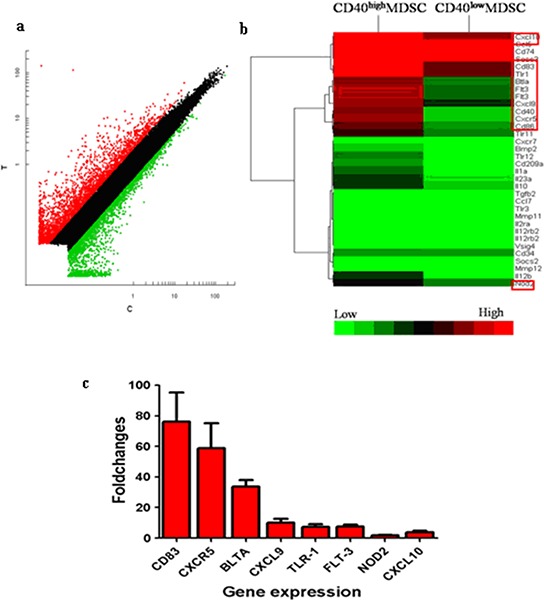
CD40^high^ and CD40^low^ MDSC presented distinct gene expression profiles Gene expression profiles of CD40^high^ MDSC and CD40^low^ MDSC were examined by microarray analysis. **a.** Scatter plot of microarray data showing genes that were more than two-fold upregulated (red), less than two fold changed (black) or downregulated by more than two fold (green) in CD40^high^ compared with CD40^low^ MDSC. **b.** Heat map representation of microarray data on genes of interest. Expression levels are indicated on a color scale where red represents higher expression and green lower expression. **c.** qRT-PCR analysis of eight genes comparing CD40^high^ and CD40^low^ MDSC expression levels. The expression level of each target gene relative to an internal control (β-actin) was determined using the 2^−ΔΔCT^ method. The fold change between the two cell groups is presented.

To explore the potential biological functions associated with the CD40^+^MDSC, we stained single cells dissociated from MFC tumors with fluorophore-conjugated CD11b, Gr-1 and CD40 antibodies (Figure 2) and sorted CD11b^+^Gr-1^+^ MDSC into CD40^high^ and CD40^low^ MDSC groups. Next, we compared the gene expression profiles of these two groups by microarray analysis (Figure 3). The microarray analysis showed that 1872 genes were differentially expressed (more than a two-fold change) between the two groups. Among the differentially expressed genes, 1308 were upregulated and 564 were downregulated in CD40^high^ MDSC when compared with CD40^low^ MDSC (Figure 3a). Heat map analysis of distinct functional groups (Figure 3b) showed that T-cell immunosuppression related genes were significantly up-regulated in CD40^high^ MDSC, including CD83, CD86, Toll-like receptor (TLR)1, TLR11, TLR12, B and T lymphocyte attenuator (BTLA), nucleotide oligomerization domain-2 (NOD2) and chemokines and chemokine receptors including CXCR5, CXCL9, CXCL10 and Fms-like tyrosine kinase 3 (FLT-3). The highest upregulations were observed for CXC5, CD83 and BTLA. To confirm the microarray data, we performed qRT-PCR on eight of the upregulated genes closely associated with MDSC function: CD83, CXCR5, BTLA, CXCL9, TLR1, FLT3, NOD2 and CXCL10. All of these genes exhibited higher expression levels in CD40^high^ MDSC when compared with CD40^low^ MDSC (Figure 3c).

### CD40 is essential for CXCR5 expression in MDSC

**Figure 4:**
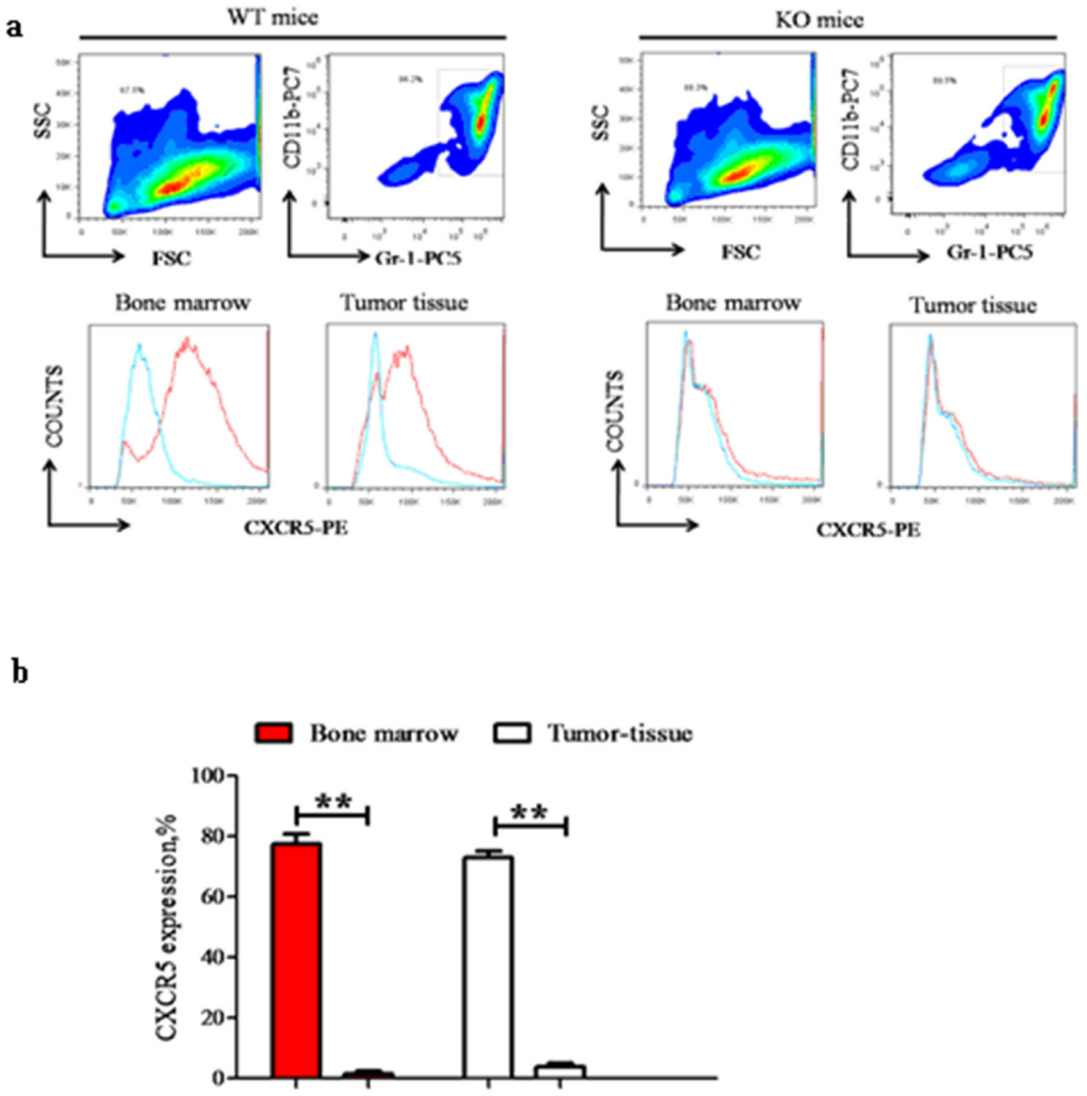
CD40 is essential for CXCR5 expression in MDSC CXCR5 expression in bone marrow or tumor tissue MDSC from WT (left panels; *n* = 6) and KO (right panels, *n* = 6) mice were examined using flow cytometry. **a.** Representative flow cytometry images demonstrating the gating strategy. The red line in the lower panels was from PE-conjugated anti-CXCR5 antibody, while the blue line was from the PE-conjugated isotype-matched IgG control sample. **b.** Quantification of CXCR5^+^% cells in different groups. **p < 0.01, comparing the same tissue from WT versus KO mice.

Among the chemokines and chemokine receptors that were differentially regulated between CD40^high^ and CD40^low^ MDSC, CXCR5 had the most significant difference in expression. To investigate the role of CD40 in CXCR5 expression regulation, we compared CXCR5 expression in bone marrow and tumor tissues from the MDSC of WT and KO mice. In both the bone marrow and tumor tissues of WT mice, a similarly high percentage of MDSC were positive for CXCR5 expression (77.33 ± 3.29% in bone marrow and 72.93 ± 2.05% in tumor tissue; *p* > 0.05; Figure 4). However, in KO mice CXCR5 expression was almost non-detectable in MDSC from the same tissues. These results suggested that CD40 is essential for CXCR5 expression in MDSC.

### The CXCR5-CXCL13 axis regulates CD40^+^MDSC migration toward MFC cancer cells

**Figure 5:**
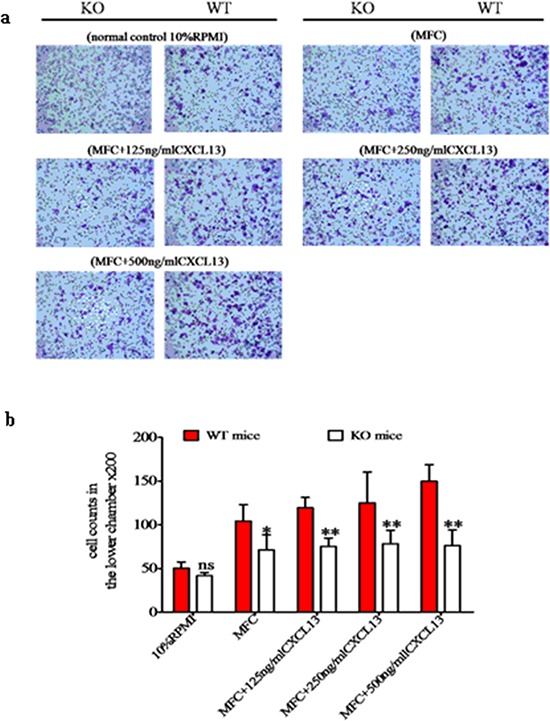
The CXCR5-CXCL13 axis regulates the directional migration of CD40^+^ MDSC towards MFC cancer cells The migration behavior of MDSC isolated from WT or KO mice towards MFC were examined using transwell migration assays in the absence or presence of CXCL13 at the indicated concentrations. As a baseline control, 10% RPMI medium was used. **a.** Representative microscope images of crystal violet stained migrated cells (200 ×). **b.** Cell migration quantification under different conditions. *p < 0.05 and **p < 0.01 compared with WT MDSC under the same migration conditions.

Because the chemokine receptor CXCR5 and its ligand CXCL13 are unique and exclusive to each other, we assessed the role of CXCR5-CXCL13 signaling in MDSC migration towards cancer cells. Using transwell migration analysis, we observed that MDSC from WT and KO mice had comparable migration capacities under baseline conditions (no cancer cells or exogenous CXCL13). We observed 50.2 ± 7.2 migrated cells per field for WT MDSC and 41.7 ± 3.8 for KO MDSC ( *p* > 0.05). In the presence of MFC cells, MDSC from both WT and KO mice exhibited higher migration activity. However, a more robust increase was observed in WT mice (104.2 ± 37.9), and this migration activity was significantly higher than the MDSC migration level observed for KO mice (61.2 ± 17.3, *p* < 0.05). Furthermore, the number of WT MDSC that migrated also increased in a dose-dependent manner (119.4 ± 11.8, 125.2 ± 35.1 and 149.8 ± 18.8) with the addition of increasing concentrations of CXCL13 (125, 250 and 500 ng/mL, respectively). This migration rate was significantly higher than the migration rate of KO MDSC under the same conditions (75.4 ± 9.5, 78.2 ± 15.6 and 76.2 ± 14.8), which did not change dramatically from the migration rate of KO MDSC in the presence of MFC alone (*p* > 0.05, Figure 5a and 5b). These results suggested that the CXCR5-CXCL13 signaling axis regulated the directional migration of WT MDSC toward cancer cells, but that the signaling axis had no effect on KO MDSC migration.

**Figure 6:**
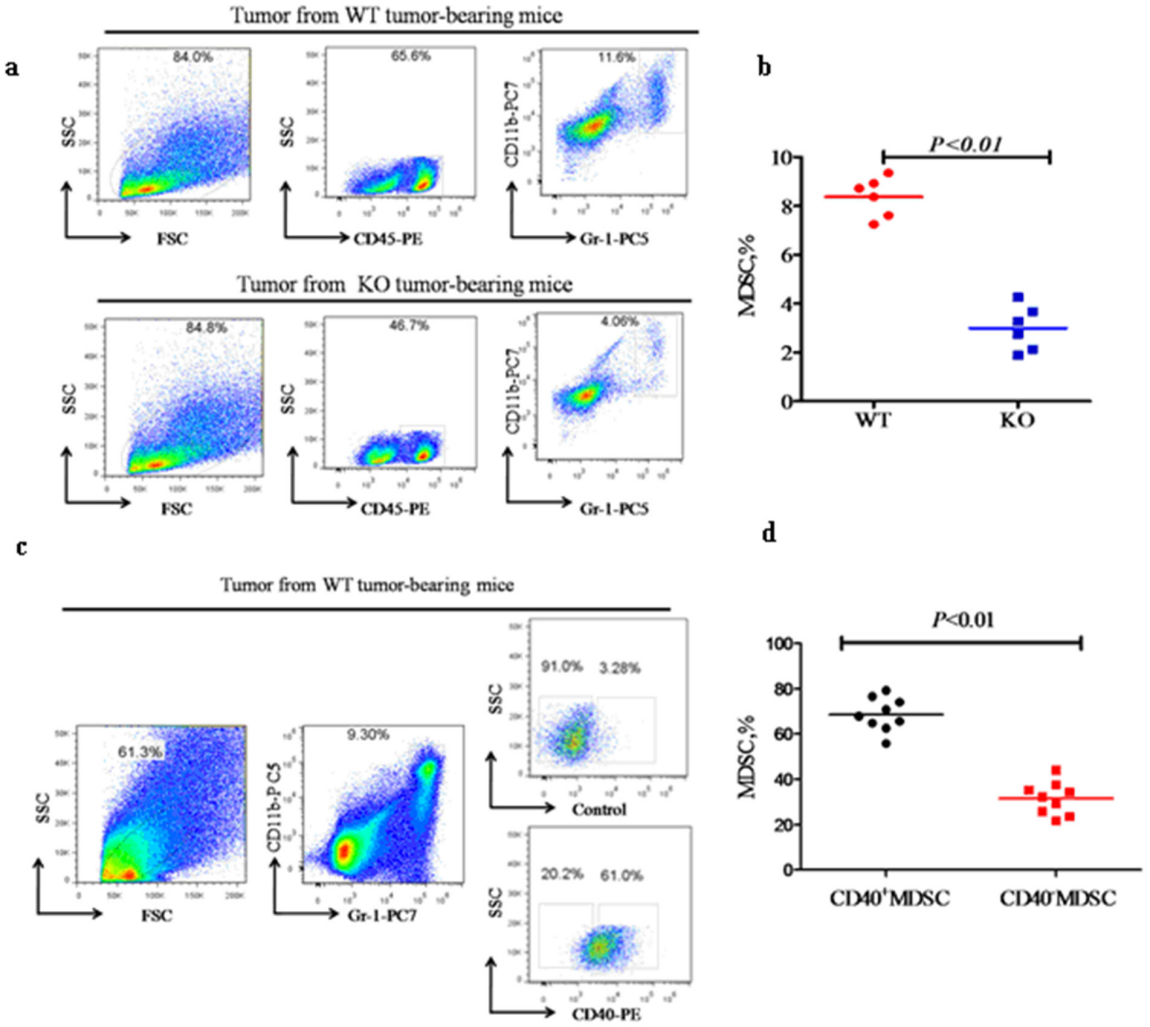
CD40 expression is associated with increased MDSC recruitment to tumor tissues Flow cytometry analysis of the percentage of CD11b^+^Gr-1^+^ MDSC (MDSC%) within tumor tissues from WT (*n* = 6) or KO (*n* = 6) mice **a** and **b.**, and the CD40^+^ versus CD40^−^MDSC subsets within WT tumor tissues (**c** and **d.** *n* = 9). a) Representative flow cytometry images demonstrating the gating strategy and a higher MDSC% in tumor tissues from WT mice when compared with KO mice. b) Quantification of the MDSC% determined in a. c) Representative flow cytometry images demonstrating the gating strategy and a significantly higher percentage of CD40^+^ than CD40^−^ MDSC in WT tumor tissue. d) Quantification of the flow cytometry data shown in c.

In addition to *in vitro* migration, we also quantified MDSC recruitment *in vivo*. The percentage of tumor-infiltrating MDSC was significantly higher in WT mice when compared with KO mice ( 8.37 ± 0.81% versus 2.96 ± 0.92%; *p* < 0.01; Figure 6a and 6b). Further analysis indicated that the CD40^+^% of tumor-infiltrating MDSC in WT mice ( 68.58 ± 7.35) was significantly higher than the CD40^−^% (31.52 ± 7.18; *p* < 0.01; Figure 6c and 6d). This indicated that CD40^+^ MDSC are preferentially recruited to and accumulated in tumor tissue. This correlates with the CXCR5 expression in these cells (Figure 4) and suggests a critical role for the CXCR5-CXCL13 axis in the recruitment of MDSC to cancer.

### CD40 KO is associated with significantly delayed tumor growth

**Figure 7:**
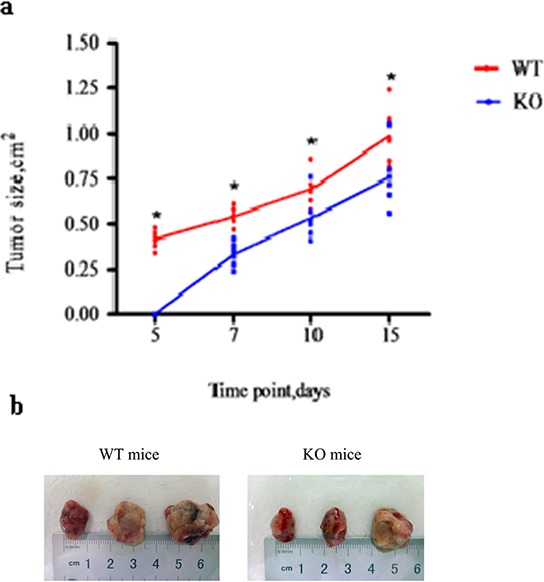
CD40 KO is associated with significantly delayed tumor growth **a.** MFC were injected into WT (*n* = 6) and KO (*n* = 6) mice. Tumor growth was measured and is presented as tumor size at the indicated post-MFC inoculation time points. *p < 0.05, compared with tumors from CD40 KO mice. **b.** Fifteen days after MFC inoculation, the mice were sacrificed and tumors were excised and photographed. Tumors from WT mice were larger than tumors from KO mice. WT tumors were also irregularly shaped, with rough surfaces and ulcers.

To examine the biological significance of the reduced MDSC recruitment observed in KO mice (Figure 6a and 6b), we monitored tumor growth in WT and KO mice. Tumor growth was significantly delayed in KO mice. Tumors could be detected in WT mice as early as five days after MFC inoculation, but not until seven days in KO mice. When examined at days 5, 7, 10 and 15 after MFC inoculation, KO mouse tumors were significantly smaller than WT tumors (*p* < 0.05, Figure 7a). When tumors were examined on day 15, the KO mouse tumors were well confined in smooth capsules, but the tumors from WT mice were irregular, with ulcers and a rough surface, indicating active tumor invasion ( Figure 7b).

## DISCUSSION

In this study, we identified a novel mechanism by which CD40 promotes MDSC-mediated immune invasion and stimulates tumor growth by controlling the expression of chemokine receptor CXCR5 on MDSC, and modulating the recruitment of CD40^+^ MDSC that depends on CXCR5-CXCL13 signaling.

Chemokines are the key regulators of immune cell trafficking in various biological processes and significantly contribute to the initiation and execution of immune responses. Studies have shown that effector T cells lacking critical chemokines exhibit deficient migration and activation, and that this results in compromised anti-tumor T cell activity and enhanced tumor invasiveness and metastasis [21]. Abnormal chemokine upregulation within the tumor microenvironment stimulates tumor growth by inducing immune evasion [22]. High CXCL9 and CXCL10 levels inhibit cytolytic CD8^+^ T cell expression of CXCR3 in hepatocellular carcinomas, and this, in turn, limits lymphocyte recruitment and tumor defense [23]. Kitamura et al. reported that CCR1 and CCL9 crosstalk contributes to the colorectal cancer infiltration of CD11b^+^CD34^+^ immature myeloid cells and subsequent tumor invasion [24]. Huang et al. showed that CCR2-CCL2 signaling mediates CD115^+^Gr-1^+^ MDSC recruitment to cancer tissues. Targeting MDSC expression of CCR2 led to the downregulation of other chemokine receptors in MDSC, such as CCR1, CCR5 and CXCR4, supporting the pivotal role of CCR2-CCL2 signaling in *in vivo* MDSC migration and tumor growth [25]. The variety of chemokines identified by these studies suggests that the subsets of MDSC preferentially induced and recruited to various tumor tissues may vary with the tumor types and chemokines involved.

In our previous study, we observed that CD40 was highly expressed in gastric tumor MDSC. During tumor progression, CD40 levels decreased, and this decrease was associated with an increased number of MDSC in the tumor. This implied that CD40 downregulation contributed to MDSC accumulation by enhancing the apoptotic resistance of MDSC [26]. Weiss et al. showed that IL-2 and anti-CD40 immunotherapy reduced CXCL5 expression and the accumulation of MDSC within the renal cell carcinoma microenvironment [27]. CD40 was significantly upregulated in the MDSC of patients with breast or prostate cancers. CD40 contributes to immune suppression through CD40L interaction by stimulating Treg expansion and upregulating PD-1 expression in CD4^+^ T cells [28]. These studies suggest that CD40 exploits multiple mechanisms to regulate MDSC recruitment and tumor functions.

In this study, we showed that in the percentage of CD40^+^ MDSC was significantly higher in xenograft tumors derived from gastric, lung and prostate cancer cells when compared with spleen tissue from the same mice. This result suggests a potential role for CD40 in recruiting MDSC to these three types of tumors. To reveal the underlying mechanisms, we compared the gene expression profiles of CD40^high^ and CD40^low^ MDSC isolated from gastric xenograft tumors. We identified 1872 differentially expressed genes (genes with more than two fold expression level changes between the two cell populations), including 1308 upregulated and 564 downregulated genes in CD40^high^ MDSC when compared with CD40^low^ MDSC. CD83, CXCR5, BLTA, CXCL9, TLR-1, FLT-3, NOD2 and CXCL10 were among the most upregulated genes, suggesting that CD40 may induce immune evasion by inhibiting T cell expansion and MDSC recruitment [29, 30]. Interestingly, several of the examined chemokines and chemokine receptors that had previously been suggested to regulate MDSC trafficking behavior, such as CCR2, CXCR4 and CCR5, were not significantly up- or downregulated between CD40^high^ and CD40^low^ MDSC (data not shown). This implies that the higher MDSC recruitment and accumulation observed in WT xenograft tumors may be mediated by a novel chemokine-chemokine receptor signaling mechanism.

One previous study reported that CXCL13 and CXCR5 were unique and exclusive to each other, and that their signaling axis controlled B lymphocytes targeting of follicles in secondary lymphoid organs, and, furthermore, that this signaling axis was essential for maintaining lymphatic system homeostasis [31]. However, the function of the CXCR5-CXCL13 axis in MDSC recruitment is not known. Our study revealed that CD40 modulates CXCR5 expression in MDSC, and that CXCR5 is sufficient to drive CD40^+^MDSC chemotaxis *in vitro*. *in vivo* analysis consistently showed a significantly higher accumulation of MDSC in gastric tumors from WT mice than from KO mice, and almost 70% of the tumor-infiltrating MDSC from WT mice were CD40 positive, supporting the hypothesis that CD40 signaling plays an important role in MDSC accumulation in gastric tumors. As a functional consequence of this newly identified mechanism and of other potential actions by CD40, tumor growth was significantly suppressed in CD40 KO mice when compared with WT mice.

In summary, we showed that CXCR5 is a novel target of CD40. Our results indicate that CD40 regulates the recruitment and accumulation of MDSC in gastric cancer by controlling CXCR5 expression in MDSC. Therefore, targeting CD40 or CXCR5-CXCL13 signaling may provide a novel cancer therapy strategy. Because CD40^+^ MDSC accumulates in gastric, lung and prostate xenograft tumors, it would be interesting to examine whether the CXCR5-CXCL13 axis is equally important for MDSC recruitment in other cancer types. Further investigation of the other genes that are differentially expressed between the CD40^high^ and CD40^low^ MDSC may lead to the discovery of other novel CD40 related mechanisms.

## MATERIALS AND METHODS

### Cell lines and experimental animals

Mouse forestomach carcinoma cells (MFC), Lewis lung carcinoma cells (LLC) and prostate carcinoma cells (RM-1) were purchased from the Cell Bank of the Shanghai Institutes for Biological Sciences (Shanghai, China). All cells were cultured in RPMI-1640 medium (Gibco-BRL, Carlsbad, CA, USA) supplemented with 10% fetal bovine serum (FBS).

Wild type C57BL/6 mice (WT; six to eight weeks old) were purchased from the Shanghai Laboratory Animal Center (Shanghai, China). Syngenic age-matched CD40 knockout mice (KO) were purchased from the Jackson Laboratory (Maine, USA). All mice were housed in a pathogen-free facility with a 12:12 h light-dark cycle at 27 ± 2°C and 45 to 55% relative humidity. Food and water were provided ad libitum. All animal experiments were approved by the Institutional Animal Care and Use Committee of Soochow University (Suzhou, China).

### Establishment of the xenograft mouse model

Cancer cells growing in the log phase were harvested and single-cell suspensions were prepared at 5 × 10^7^/mL (for MFC) and 5 × 10^6^/mL (for LLC and RM-1). The cell suspensions were subcutaneously injected into the inguinal region of the mice (200 μL/mouse). Mice were sacrificed when the tumors had grown to approximately one centimeter in diameter, and the spleen and tumor tissue were removed for MDSC isolation.

To compare MFC tumor growth in WT versus KO mice, MFC cells were subcutaneously injected into the inguinal region (200 μL/mouse). Tumor length (L) and width (W) were measured every two to three days. Tumor volume (V) was calculated according to the following formula: V = 1/2 × L × W^2^.

### MDSC isolation

MDSC were isolated from single-cell suspensions of various mouse tissues using an MDSC isolation kit (Miltenyi Biotec, San Diego, CA, USA) according to the manufacturer's instructions.

### Flow cytometry analysis

The following cell staining antibodies were purchased from Biolegend (San Diego, CA, USA): PC7-conjugated anti-mouse Gr-1 antibody, PC5-conjugated anti-mouse CD11b antibody, PE-conjugated anti-mouse CD40 antibody, FITC-conjugated anti-mouse CD45 antibody (Biolegend, San Diego, USA). PE-conjugated CXCR5 antibody was purchased from Miltenyi. Flow cytometry was performed using a Cytomics FC 500 flow cytometer (Beckman Coulter, Brea, CA USA).

### Microarray analysis

CD40^high^ and CD40^low^ MDSC were sorted using FACS Aria (BD Biosciences, San Jose, CA, USA), and total RNA was isolated using Trizol (Invitrogen, Carlsbad, CA, USA) according to the manufacturer's instruction. Total RNA was reverse transcribed into double-stranded cDNA using CbcScript enzyme (CapitalBio, Beijing, China). Next, cRNA was synthesized from cDNA using a T7 enzyme mix (Takara Bio, Dalian, China) and purified using an RNA Clean-up Kit (MACHEREY-NAGEL, Düren, Germany) according to the manufacturer's instructions. Newly synthesized cRNA was reversed transcribed into cDNA using random primers and CbcScript II enzyme (CapitalBio). Finally, transcription products were purified using the Nucleospin^®^ Extract II Kit (MACHEREY-NAGEL), and fluorescently labeled with Cy5- or Cy3-dCTP using the Klenow enzyme (TakaRa, Mountain View, CA, USA).

Gene expression profiles were examined by CapitalBio using a Mouse (V2) Gene Expression Microarray 8 × 60K chip (Agilent, Santa Clara, CA, USA). Microarray data were analyzed using GeneSpring GX software (Agilent). Genes showing more than two-fold absolute changes between CD40^high^ and CD40^low^ MDSC were defined as differentially expressed.

### Quantitative real-time PCR (qRT-PCR)

Total RNA from CD40^high^ and CD40^low^ MDSC cells was reverse transcribed into double-stranded cDNA using CbcScript enzyme (CapitalBio), and qRT-PCR was performed using the primers listed in Table 1. The thermal cycling conditions were as follows: denaturation at 95°C for 10s, then 39 cycles of 95°C for 5s and 60°C for 30s. Target gene expression levels were normalized against β-actin, and the results were calculated using the 2^−ΔΔCT^ method, as previously described [19].

**Table 1:** Primer sequences used for quantitative real-time PCR

Target Gene	Forward Primer	Reverse Primer
CXCL10	CGTCATTTTCTGCCTCATCCT	ATTTCTTCCTGCTTCCCTCTTCT
BTLA	CAGGACCATCCACCATGGAA	TGTCCTGGAACTGGCTGGAA
CXCR5	GGACATGGGCTCCATCACATAC	CACAGGCATGAATACCGCCTTA
CXCL9	CCGAGGCACGATCCACTACA	AGTCCGGATCTAGGCAGGTTTG
CD83	AAGTACAGGGCAGAAGCTGTGTTG	AAGCTTGTTCCGTACCAGGTTTAGA
FLT3	GCACGTCTTGCGAAACCATC	CAGAGCCCAATGGTCGCATA
TLR1	GGTTCCGTGATGCACAGCTC	CAGAGCATTGCCACATGGGTATAG
NOD2	ACCATGTAGAAGCCATGCTGGAG	CTTCACCGCAGCGAGATCAA
ACTB	GTGACGTTGACATCCGTAAAGA	GCCGGACTCATCGTACTCC

### Transwell migration assay

Transwell migration assays were performed as previously described [20]. Briefly, MFC cells (1 × 10^4^ cells in 500 μL RPMI-1640 medium containing 0.5% FBS) were mixed with recombinant mouse CXCL13 (R&D, Minneapolis, MN, USA) to final concentrations of 0, 125, 250 and 500 ng/mL and seeded into the lower wells of 24-well transwell plates (5-μm pore size; Corning, Union City, CA, USA). Flow-sorted CD40^high^ or CD40^low^ MDSC were resuspended in RPMI-1640 medium containing 0.1% FBS at a final concentration of 1× 10^5^/mL and loaded on top of the filter membranes of the transwell insert (100 μL/well). After an eight-hour incubation at 37°C with 5% CO_2_, the transwell insert was removed from the plate and a cotton-tipped applicator was carefully used to remove the media and non-migrating cells from the top of the membrane without damaging it. Next, the transwell insert and migrated cells were placed in anhydrous ethanol for 15 minutes for cell fixation. After allowing ethanol evaporation at room temperature, the transwell insert was stained in a 0.1% crystal violet solution for 30 min. After the excess crystal violet was removed, the migrated cells were imaged under an inverted microscope (200 ×). Cells were counted in five different fields of view (upper, lower, right, left and center) to determine the average number of cells that migrated through the membrane. Three independent experiments were performed for each condition.

### Statistical analysis

Flow cytometry analysis was performed using Flowjo software (Flowjo, Ashland, OR, USA). All statistical analysis was performed using Prism software (GraphPad, La Jolla, CA, USA). The statistical differences were analyzed using a *t*-test and one-way or two-way analysis of variance. Quantitative data were presented as means ± SEM. A two-sided *p* value of < 0.05 was considered statistically significant.
